# Shortcomings of services for persons with severe and persistent mental health challenges: a qualitative study of service users and family carers

**DOI:** 10.3389/fpsyt.2024.1341248

**Published:** 2024-02-14

**Authors:** Anton Isaacs, Caroline Lambert, Sharon Lawn, Anna Dyer

**Affiliations:** ^1^School of Rural Health, Monash University, Warragul, VIC, Australia; ^2^Tandem Inc., Abbotsford, VIC, Australia; ^3^RMIT University, Melbourne, VIC, Australia; ^4^Lived Experience Australia, Brighton, SA, Australia; ^5^Flinders University, Adelaide, SA, Australia; ^6^Latrobe Regional Hospital, Traralgon, VIC, Australia

**Keywords:** mental health services, healthcare quality, access and evaluation, health services needs and demand, caregivers, patients

## Abstract

**Introduction:**

The opinions of service users and carers are crucial to identifying ways to innovate and implement system change. This study aims to explore the views and experiences of service users and carerson the services they have used for their mental health challenges and their suggestions for service reform.

**Methods:**

Twenty participants (15 carers and 5 service users) were interviewed for the study.

**Results:**

Eight categories emerged from the data. They were: Several gaps in the system, Barriers to accessing services, Services are not fit for purpose, Services operate in isolation, System is not person focused, Service users and carers are treated poorly, Services are overloaded and under resourced and Recommendations for service reform. Respondents reported that a persistent lack of funding and resources for mental health services was a main cause of these shortcomings. Respondents also noted that innovations were needed to re-orient services to enable continuity of care, and training of mental health professionals was needed for a better understanding of the needs of service users and their carers.

**Discussion:**

Additional research is needed with larger and more diverse samples to further explore these findings.

## 1 Introduction

The burden of mental disorders worldwide is the largest of all health problems. They account for 13% of the total burden of disease and 32.4% of years lost due to illness or disability (1). The more severe forms of mental illness such as bipolar disorders, major depression, schizophrenia, and anorexia nervosa typically fall under the umbrella of severe and persistent mental illness (SPMI) (2). Persons are said to have SPMI if they have experienced severe and disabling mental health symptoms for a long period of time (3). They often have a combination of disorders with significant global impacts on the rest of the domains of life that render their care more challenging (2). In this paper, we will use the term severe and persistent mental health challenges [SPMHC] instead of SPMI since most of the needs experienced by individuals with this condition are non-clinical in nature (4).

Australia’s mental health system encompasses a wide range of clinical and non-clinical services (5). Perhaps the most prominent of them are the area based clinical mental health services, mental health community support services which are non-clinical services provided by non-governmental organizations, private mental health services (Psychiatrists and psychologists) and the National Disability Insurance Scheme (NDIS). Mental Health Community Support Services also run adult prevention and recovery care (PARC) services which are community-based, short-term supported residential services for people experiencing mental health problems, but who do not need a hospital admission (6).

The NDIS has a remit to support persons with physical and psychosocial disabilities. It focuses on helping such individuals identify their needs and provides them with funding to access those services (7). Clinical mental health services which are mostly designed to reduce symptoms and improve function, find it difficult to provide adequate care for persons with SPMHC (8). This is because individuals with these conditions have multiple needs such as those related to accommodation, daytime activities, employment or volunteering and a lack of information on their condition (4). An earlier study of submissions to a government inquiry into the provision of mental health services in Australia reported challenges in accessing mental health care, and that care varied in quality and was poorly coordinated with services from other sectors, such as housing (9). More recently, a report of a survey of 2441 Australians showed that 49% of Australians faced barriers to accessing mental health supports (10). The report also stated that carers faced barriers to accessing mental health supports (including financial barriers), and were left feeling that they had to solve their problems alone (10).

Informal carers are typically close family members or relatives and are recognised as a fundamental resource for mental health service provision, as well as a rich source of expertise through experience (11). While informal carers are often seen by government services as a free source of labour propping up an under resourced system, we know that caring responsibilities have a significant, negative impact for those providing support. When compared to the general population, carers have almost twice the risk of psychological distress, half the rates of positive wellbeing, and 4-5 times the rate of loneliness than the mainstream population as well as high rates of financial distress (12, 13).

There are also several issues with the NDIS that make it difficult for people with SPMHC and their informal carers to access support. These include complex application processes, the inability of the service to address changing needs and the lack of available services (14). The National Framework for Recovery-oriented Mental health services has been developed as a guide for mental health services across Australia and recommends that services be person centric and holistic, promote a culture and language of hope and optimism, support personal recovery, take action on social inclusion and social determinants, establish organizational commitment and develop workforce to implement these actions (15). However, ten years after this framework was published, ground realities do not appear to have changed. The COVID -19 pandemic and its consequent rise in need for services has worsened the situation. The Royal Commission into Victoria’s Mental Health System has highlighted the broad issues affecting Australia’s mental health system (16). The Commission reported that access to services is difficult and inequitable and that the system is driven by crisis. The Commission also reported that there is an overreliance on medications and less emphasis on personal recovery, that carers and families are left out of care and that services are not integrated (16).

Learning from the experiences of service users is crucial to identifying ways to innovate and implement system change since they live with the daily consequences of SPMHC on their lives and directly experience the impacts of service and system reforms (17). The Global Mental Health slogan, ‘Nothing about us without us’ directly speaks to the importance of their involvement in the design and implementation of services for persons with SPMHC and their families (18). This is also a human rights issue with the need to incorporate service user and carer input into existing services gaining traction worldwide (19). The Australian Commission on Safety and Quality in Health Care has issued a Partnering with Consumers Standard to create organisations where consumers are partners in planning, design, delivery, measurement and evaluation of systems and services (20). However, to what extent this standard is adopted is unknown. Carers (or caregivers) are also key contributors to this endeavor and need to be recognised and acknowledged for the important role they play in the care of the persons with severe mental illness (21). They are critically important to the recovery of the persons they care for (22) and experience multiple challenges (23).

This study aims to explore the views and experiences of service users and carers on the services they have used for their mental health challenges and their suggestions for service reform.

## 2 Methods

### 2.1 Study design

This was a qualitative study of service users and carers that was underpinned by Qualitative Description (QD). QD was first introduced by Sandelowski (24) and aimed to present a straight description of an experience or event (25). Hence the researcher avoids any excessive interpretation of the data (24) and the resulting information is hence grounded in the existing cultural and environmental contexts. QD is therefore useful when the research is aimed at improving healthcare interventions (26) and is particularly useful when obtaining information for service providers and policy makers. QD has been used in similar studies previously (27, 28).

Apart from the lead investigator, the research team includes 1. A professor of public health with a lived experience of mental health problems who is also a representative from Lived Experience Australia, a national advocacy organisation for consumers, families and carers in all mental health settings [SL] (29), 2. A mental health academic who is also a carer with a lived experience of mental health problems and representative of Tandem Inc., the Victorian peak body representing family, carers and supporters of people living with mental health challenges [CL] (30) and 3. A lived experience worker at a regional mental health service [AD]. This has ensured that the language used in this paper was inclusive and the conclusions drawn did not in any way shift from the actual views and experiences of service users and carers.

### 2.2 Participant recruitment

An invitation to participate, together with the Explanatory statement and Consent form was sent out in the news bulletins of Lived Experience Australia and Tandem Inc. These were coordinated by authors SL and CL respectively. Those who were interested in participating contacted the lead researcher to choose a suitable date and time for an interview. Sample size for qualitative studies where participant experiences are more similar than different, typically lie in the 12-13 range for data saturation (31). We aimed to recruit 10 service users and 10 carers.

### 2.3 Data collection

Semi-structured interviews were conducted over the phone or videoconference with participants over the age of 18 with a self-reported history of severe mental health challenges. Interviews lasted between 30 and 45 minutes, were audio recorded and professionally transcribed. Diagnoses were recorded as mentioned by participants. Transcripts were coded using an alpha-numeric code (e.g. P4 Consumer). Participants were reimbursed for their time with gift vouchers worth AUD 40. The tentative interview schedule for service users and carers is given in **Box 1**. Ethics approval for this study was obtained from Monash University Human Research Ethics Committee (Project ID: 35429; Approval date: 19/09/2022).

Box 1 **Tentative interview questions for service users and carers.**

Service users

1. Age/ Gender /cultural identity/ highest education / occupation if any / living alone or otherwise / Housing / presence of a carer / Diagnosis / Duration of illness / number of hospitalizations.
2. Could you share your experiences with mental health services from the time you started accessing them?
3. What worked well? How?
4. What did not work well? Why not?
5. What if any suggestions do you have for the improvement of services for people with severe mental health challenges?

Carers

1. Age/ Gender / cultural identity/ highest education / other occupation if any / Relationship if any with the person being cared for/ Duration of caring responsibilities.
2. Could you share your experiences with mental health services from the time you started accessing them?
3. What worked well? How?
4. What did not work well? Why not?
5. What if any suggestions do you have for the improvement of services for people with severe mental health challenges?

### 2.4 Data analysis

Data were analysed inductively. Chunks of data that captured a rich qualitative aspect of the phenomenon were assigned codes. Once all data were coded, codes were refined and finalised either by combining, separating or renaming them to ensure that they accurately reflected the data they represented. Data were initially coded by the lead author. Three other authors (SL CL and AD) independently reviewed the coded data and made modifications as needed. As a result of this process, two participants were contacted a second time for further clarification. Codes were finalised once there was agreement between all four authors. The lead author then grouped the codes into categories that reflected a broader phenomenon. Categories with their included codes were reviewed by the three other authors who recommended changes where necessary. Categories with their included codes were finalised after agreement between the four authors. As per Creswell’s suggestion, we decided to conduct member checking with ‘interpreted pieces such as themes and patterns emerging from the data rather than the actual transcripts’ (32). Accordingly, a copy of categorized and coded data was sent to all participants for comments. One participant made modifications to their contribution having recognised their words in the data. An audit trail (32) of every step of the analysis has been maintained. Categories, and their representative quotes are presented and discussed below. The NVivo software was utilized to aid data analysis.

## 3 Results

A total of twenty participants (15 carers and 5 service users) were interviewed for the study. All but one carer was female. Thirteen of the 15 carers were mothers who cared for their own adult children and more than 50% had been in the caring role for over 10 years. Among the service users, three were male, one was female and one was a transgender man. Among the service users, 3 of the 5 had multiple diagnoses and 4 of the 5 had been hospitalized for their illness more than 6 times. Further details of demographic characteristics of participants are given in **Table 1**.

**Table 1:** Characteristics of carers and service users (n=20).

Characteristics of carers (n=15)	Frequency	Characteristics of Service users (n=5)	Frequency
**Age range**		**Age range**	
31-40	1	21-30	2
41-50	3	31-40	1
51-60	3	41-50	0
61-70	5	51-60	1
71-80	3	61-70	1
**Gender**		**Gender**	
Female	15	Male	1
**Culture**		Female	3
Australian	12	Trans man	1
Rainbow family	1	**Culture**	
Indian Australian	1	Australian	5
Greek Australian	1	**Education**	
**Education**		Doctorate	1
Masters	2	Bachelor	1
Diploma	5	Certificate	2
High school	3	High school	1
Certificate	5	**Occupation**	
**Occupation**		Optometrist	1
Physiotherapist	1	Support coordinator	1
Pathology collector	1	Restaurant work	1
Sales and marketing	1	Manager in health service	1
Bookkeeper	1	Arborist	1
Nurse	1	**Diagnosis**	
Project officer	2	Anxiety/depression	2
Teacher	1	Borderline Personality Disorder, Bipolar disorder	2
Retired	2	Generalised anxiety disorder, Major depressive disorder, and Borderline personality disorder	1
Finance industry	1	**Years living with mental illness**	
On carer payment	4	1-10	1
**Diagnosis of the person cared for**		11-20	2
Anxiety/depression	2	21-30	1
Schizophrenia	3	31-40	1
Borderline Personality Disorder	1	**Number of hospitalisations**	
Eating disorder	1	1-5	2
Intellectual disability	1	>6	3
Anxiety disorder, Schizophrenia	1	**Has a carer**	
Anxiety disorder, Depression, Autism spectrum disorder	1	Yes	3
Schizoaffective disorder, Autism Spectrum Disorder,?Attention-deficit/hyperactivity disorder	1	No	2
Generalised anxiety disorder, Obsessive Compulsive Disorder, Auditory Processing Disorder., Sensory processing disorder. Oppositional defiant disorder	1		
Borderline Personality Disorder, Anxiety, Depression	1		
Bipolar disorder, Borderline Personality Disorder	1		
Bipolar Disorder, Complex Post-traumatic Stress disorder	1		
Years caring for person with mental health challenges			
1-10	7		
11-20	5		
21-30	3		
Relationship to person with mental health challenges			
Mother	13		
Husband	1		
Aunt	1		

Eight categories emerged from the data. They were: Several gaps in the system, Barriers to accessing services, Services are not fit for purpose, Services operate in isolation, System is not person focused, Service users and carers are treated poorly, Services are overloaded and under resourced and Recommendations for service reform. Each of the categories and their representative quotes are described below.

### 3.1 Several gaps in the system

This category relates to participant views on the various gaps in the mental health system that resulted in no services available for certain groups of individuals. For instance, one respondent identified that there was no service for those who, owing to their illness remained isolated at home and did not reach out for help.

I think, probably the biggest thing for me is that mental health services are unable to offer a solution to the gap that they have created, … through their framework in which they work. They cannot find the people who are at home isolated and refuse to reach out for help. There is no method to resolve that gap, I call it the great gap in service offerings … there is no solution … they don’t want to talk to me, because I’m the mother. – P3 Carer.

Individuals with PTSD did not get the help they needed unless they were acutely ill as a service user explained.

You know dealing with PTSD every day is a challenge … They’re not getting the best. They’re in the middle, they’re not presenting in psychosis. They’re not bad enough for psychosis, but they so desperately need help. That’s a gap in the system. Don’t let it become psychosis, don’t let them become so broke, that they can’t be fixed. Fix them back here. – P11 Consumer.

Similarly, another service user indicated that there were no services available to help people before they reached a point of crisis.

I think the biggest problem is the support available in prevention of a crisis situation. To prevent someone getting to a point of being suicidal or hurting themselves or, you know, overdosing, whatever it is. – P17 Consumer.

Participants also emphasized the lack of afterhours services.

I reached out because I knew I was becoming unwell. And because it was after five o’clock, the only thing I could do was call an ambulance or police. And I really had to do that. But that still took a long time. And by then I’m too far gone. And I just ended up basically looping. – P18 Consumer.

A lack of community support after discharge was another gap in the system highlighted by this carer.

When she comes out of hospital, there’s not enough community to support … but there are no beds!!! When she came out of hospital, she [would need to be] supported 24/7, she would also need dietitian input, she would need medical monitoring, she would need psychologists’ appointments. She would need a lot of support. … there’s actually no model of care for them. Has the family meant to cope? Has the family meant to keep them alive? It’s just wrong. It’s just humanely wrong. And they’re at risk and highly vulnerable [in the] community and that is known, but everyone’s ticking their boxes here. – P7 Carer.

Another carer bemoaned the lack of services for families.

I’ve spent the last two years trying to find a place that will accept not just myself and work with just me, but my children as well, because you know, my 16-year-old daughter, she’s suffered mental health her whole life. I’ve had her in and out of many therapies … Everywhere, I’ve turned to, it’s either not suitable … It only works if the parent’s gonna (sic) solve all the kid’s problems. And when you go into the mental health sector, they don’t take meeting parents into it, like I’m kind of not traded. – P14 Carer.

Finally, the lack of services for transgender people was also reported.

A lot of the services are not equipped in any way to deal with people who have complex issues or, in my case, I found that a lot of services don’t know what to do because I’m a trans man. Where do we house you? Is it male, female? Do we need to segregate you … like … you know, they don’t know. Things that might affect someone who’s transgender, the effects that being trans might have on your mental health and stuff like that. – P17 Consumer.

### 3.2 Barriers to accessing services

This category relates to the various barriers experienced by service users in accessing services. Stigma continues to be a barrier to service access as this carer describes.

We need to be more open to difference and open to what sometimes happens to people. And we need to understand that yes, sometimes people go completely bananas and do really dreadful things. But if we cast everybody into that mould, then it’s just going to be awful for people who find themselves unwell. And they won’t actually say anything. I mean, a lot of people will hide it, rather than get help. – P20 Carer.

Getting admission to a service is a long and laborious process during which time one’s illness could get considerably worse, as this participant stated.

If I needed to go back to PARC [prevention and recovery centre], I would need to contact my case manager, [then] wait till she could look at doing a referral. I’d have to have specific reasons and stuff like that for the referral; it would then be sent to PARC where they would review it, and then if they didn’t have any beds or anything, I would go on a waiting list. From that waiting list, you then have to go in and have a meeting with PARC. And then they will decide whether or not admission to PARC is appropriate for you. And then after that meeting, you wait to be contacted to be told, “Okay, we have a bed, you can come in”. But that process could take weeks. And so, if you’re at a point where you’re starting to get unwell and you’re on a path to a crisis situation, those few weeks can feel like months. – P17 Consumer.

The lack of information on services available was also a barrier.

When you come out [from hospital] you’re just on your own … it needs that continuity of care of having supports there and also supports for carers. I find that’s really lacking information more than anything as to what’s needed and what’s going on, is a big thing for carers. I feel like when I was sick last, my kids were really in the dark about what I needed and what was going on. – P8 Carer.

### 3.3 Services are not fit for purpose

In this category participants highlight a lack of fit of services to address needs of individuals with severe mental health challenges. For instance, opening times of services did not match the times when services were most needed.

I can do e-headspace for younger guys. But you know, 1 am in the morning it closes off. I don’t know if people realize but it’s definitely from midnight to five o’clock … midnight to three, mental health crashes because no one’s here. Everyone’s going to be sleeping, but I can’t sleep. My brains … you know … darting ahead. My carer, she’s out cold … don’t want to upset her. And that’s their big crisis bubble. Unless you’re significantly medicated or was so exhausted. And I think if you walk into an ED department, talk to any nursing person, that’s the awful time… 1, 2, 3 and off they go. – P12 Consumer.

A carer who also had mental health problems describes how she was exasperated, when having overcome numerous barriers reached a healthcare professional, only to be told that there was nothing that could be done for her.

How frustrating it is to sit in front of a psychiatrist and then burst into tears because you’re so exhausted, and you’ve finally got yourself there. And they’re not offering you anything. – P3 Carer.

Participants lamented that the private system was more accommodating than the public system in helping service users find treatments that worked for them.

The public system isn’t really built for extensive, long term treatment. I kind of saw people once or twice, and they’d say, you know, it’s just borderline personality disorder, you need to do DBT [Dialectical behaviour therapy]. “No medication is going to help you … and do some mindfulness”. And that’s it. Whereas, obviously, privately, you can keep coming back as often as you want. – P10 Consumer.

Due to previous experiences of having received poor care, participants reported waiting until the last minute before accessing a mental health service.

If the little supports have been in place, you know, look, “I’m really feeling unsafe, I’m feeling really vulnerable. I’ve just shut myself off. I know, when I’m about to have a wobble. But I’ll wait till the last possible minute to contact a public health service because I know, we’re not going to get much back. – P11 Consumer.

### 3.4 Services operate in isolation

This category describes how each service in the mental health system works in isolation resulting in a fragmented system of care. A carer narrates how she described her son’s condition to the service provider.

Area, mental health was saying, “Not appropriate, because he has his NDIS and it’s disability”. It was more disability as an ASD [Autism spectrum disorder] diagnosis. But I tried to explain to them. “I have a medically awkward depression. But because I’m socially isolated, I then become depressed. So, then I don’t want to get out of bed and the cycle goes backwards”. If we look at why am I depressed it’s because I’m socially isolated, but to get me more socially engaged, you need to actually deal with my depression. First get me some support around that. And then once I got my feet on the ground, you don’t have to focus on my depression. You focus on my social isolation and that was the battle. So, getting onto NDIS for mental health is extremely difficult. – P4 Carer.

Similarly, the PARC service was unprepared to manage physical health problems as this service user describes.

The last time I was in PARC, I woke up in the middle of the night in a panic attack. And I tried to get out of bed to turn the light on and I fell over and because I have arthritis and stuff like that I can break bones easier. That night, nurses were like, “Here, have some Nurofen and Panadol’. And then the next morning, I explained to them that my wrist was really swollen, I was in lots of pain. I asked if one of the doctors could see me. And the nurse came back to me and was like, “The doctor said they deal with mental health issues, not bones. You need to go to an offsite doctor to get seen”. So, then I had to wait until my dad could come and pick me up and take me somewhere to get it looked at and it turned out I had fractured my wrist. But I didn’t receive any actual care for my fractured wrist. – P17 Consumer.

Another example of the mental health service working in isolation was that general practitioners were not considered part of the mental health team. A carer who also experienced mental health problems described it.

I actually fought for my regular GP of 11 years. I see him religiously every two weeks and have done for the last four years. I fought for him to be part of the professional’s care team meeting. Because he’s just a regular GP, they don’t consider him a vital part of my mental health care team which is hilarious to me. Because he’s the one who has to do the referral for the psychologists. He’s the one who received the letter from the psychiatrists when I got my BPD [Borderline Personality Disorder] diagnosis, saying, ‘Here’s the diagnosis. I’m recommending this medication and this medication. So, can you write her a script?’ It wasn’t the psychiatrist who did the script. It’s all put back on my doctor. Yet, he’s not considered a vital part of my care team. And he’s not considered a professional who helps me manage my mental health. –P14 Carer.

A service user described how the isolation of services resulted in a lack of continuity of care.

If you put a referral out on the PHN [Primary Health Network], they don’t do functional assessments. They’re expensive to go for either. And then I find out that had I been given an OT [Occupational therapist] while I was in PARC, and that would have continued because I went on to get a [local mental health service] mental health caseworker. So it’s kind of like you’re a hot potato that they just dropped. Yeah. And you just feel like you have to start again. So yeah, no continuity at all. – P12 Consumer.

### 3.5 System is not person focused

In this category, respondents describe how the mental health system focused more on the clinical diagnosis rather than on the individual. Respondents indicated how services were not interested in listening to the realities of their lives and circumstances but were more interested in their diagnosis.

There’s no inclusion for my life, what my life is on a day to day basis, you know, the focus is on the mental illness, the diagnosis, not the person, not the reality…. [They] don’t actually listen to what struggles are…. I have two children with disabilities living with me and apparently, I’m not eligible. It’s not suitable. Why? I don’t know. – P12 Consumer.

Similarly, respondents objected that they were are discharged before they were ready to go home.

I’ve been at PARC where, you know, it’s come close to the end of my stay there because they’re like, ‘No, you will be here for seven days and that’s it, or 14 days and that’s it’. And I’ve come to the end of my stay there and I’ve been not well. There was a time where I went to them and I explained to them, ‘I am still suicidal, like, I want to hurt myself here. It’s not safe for me to go home’. And they’ve discharged me anyway. – P17 Consumer.

### 3.6 Service users and carers are treated poorly

This category highlights the prospect that patients with a psycho-social disability tend to be discriminated against by mental health services. Participants regretted that service users and carers were not properly listened to by service staff and that user’s experiences were often trivialized. For instance, a service user expressed anger and frustration when she was treated in a condescending way.

I know when I’ve been in a position of complete and utter vulnerability and as low as anybody can go, to just be absolutely broken, and have no hope … Losing hope is the worst thing that can happen. And I’ve been there … I was minimized and told to perhaps do trivial things to improve my condition. “Go for a walk, regulate your emotions, do some meditation, and mindfulness, eat healthy, go to the gym”. You know, when you’re in that position that’s just incredibly patronizing. You know, you’re not there because you want to be there. You’re not sitting in front of someone, a broken person because that’s where you want to be in life, and you want that attention. It’s, it’s pure desperation. I accessed and waited for months and months and months to see a psychiatrist. If you haven’t got that level of respect from them, you’re not going to get it from the people that work under them and the supporting psychologists and all that sort of stuff. And I think that that would have made a huge difference to my recovery. – P11 Consumer.

The same participant continued to describe how she felt discriminated against.

I think as women; this diagnosis of borderline personality disorder is so often wrongly diagnosed. Because women are often more emotional, they show those emotions and it then becomes attention seeking behavior, you get labelled with borderline personality disorder, you can kiss being treated and respected within the public health system, goodbye! It’s completely unacceptable … it’s just shocking. – P11 Consumer.

Another service user indicated that services tended to diminish them.

I’ve got a friend … she’s funny as hell, her and we get along great. They treat her like she’s five years old. Like she’s not capable … [She’s] more than bloody capable, you know, but she’s got some diagnosis. It’s sad, because that’s what takes away who we are. It takes away our importance, you know, and we fight like hell for it. but then we feel like we’re hitting dead ends, because there’s no continuity, or it doesn’t exist where we are, or we can’t access it, you know. So, it’s really, really hard when services pigeonhole us. And they don’t think outside of that box … Yeah. – P12 Consumer.

Carers were also not included or consulted in care with the responsibility put entirely on the service user.

How many people are out there as grown adults, [have] their families come in … kids that have their families included, … Where are they brought in? No mention has been made to bring in someone to talk about my recovery plan, or things like my treatment plan. Like, sometimes it’s very difficult for me to even talk, let alone think or make decisions, things like that. So, when I go to these appointments, it’s kinda like, they’re counting on me being on my own. Yeah,… It’s terrible. Absolutely terrible. – P14 Carer.

While noting some positive experiences with services, a service user stressed that those should be the rule rather than the exception.

There’s a couple of spots along the way where people have made a real difference. But that shouldn’t be called out. That should be the experience. Because that’s the role, you know, like to talk about people going above and beyond but I’m grateful for her. That’s not going above and beyond … that’s doing her job. And I think in a lot of areas, it’s become that way. – P11 Consumer.

### 3.7 Services are overloaded and under resourced

In this category participants describe how mental health services are under resourced and are hence unable to cope with the client load. A service user and carer stated this clearly when they said,

I think that there’s so many opportunities to help a person recover from chronic stages of their illness. And the public system just doesn’t have the resources or whatever to do it. – P13 carer.

You realize there is a system out there and it’s probably incredibly underfunded and under resourced. I know that case managers have very high caseloads. Okay. I think also the support for community mental health teams and stuff like that is lacking. – P17 Consumer.

The lack of resources affected patient care as this service user described.

I spent two weeks in PARC which I didn’t get anything out of. Didn’t get anything out of … I felt that the mental health nurses always had too much paperwork, instead of actually helping in that time of need, and I had a few times of need and it was kind of like, “Well hang on! we’ve spent five minutes with you. We gotta [sic] go do paperwork…” mental health is hard when you feel alone and not heard … makes it worse– P12 Consumer.

Mental health staff also begin to cut corners and as a result, important aspects of care such as follow-up are missed, as described by this carer.

The mental health workers … they struggled. And then after he was finally taken in, which was only a week. So, in a week, they obviously medicated him, they thought they’d fixed him. And once he came out, they promised to check on him each day, and make him take his medication in front of them. Well, they didn’t even turn up the first night. They didn’t have staff, didn’t ring, didn’t make contact to see if it was okay or to explain they couldn’t get there. [Everyone] thought someone else was coming. They had no idea. No follow up at all. So, was up to me to keep contacting them? They came once or twice and decided it wasn’t necessary. – P6 Consumer.

Participants also reiterated that if one could afford to consult private psychiatrists and psychologists who are quite expensive, the service was of much better quality.

I can pay to have consistency and continuity of care because I see a private psychiatrist. It’s really expensive. But it’s worth it for me but I have the capacity to do that. I don’t have to retell my story over and over and over again, and get different opinions from different people, because I’m only seeing the registrar who’s on there, you know. – P12 Consumer.

### 3.8 Recommendations for service reform

This category describes participants views on the type of reforms needed for the mental health system. A carer highlighted the need for care to be tailored to people’s needs.

I feel the system is very geared up to treat everyone in the same way; And there are assumptions that you’re going to engage with the system in a certain pattern. And I feel that there’s still need for some more culturally nuanced solutions and diverse options. So [I] feel [that] not every therapy is for everyone. People will not respond in the same way to … you know, seeing the psychologist or psychiatrist or going to a hospital. There needs to be maybe more innovative thinking going on about how the system can help different people rather than offering the same solution to everyone. – P8 Carer.

The same carer suggested a more holistic approach to care.

If I get very sick; I can’t work; I then have financial consequences. If they change my medication, I always have physical consequences from medication. I am dealing with a few physical issues that have arisen as a result of medication. So, there’s all of those more holistic things. Just looking at me as a whole, rather than me, you know, having a bit of a breakdown, and just fixing that … It’s looking at the bigger picture, the broad picture of everything that’s going on in my life, how it affects my relationships with my children, because it really does involve providing them support, as well as me. – P8 Carer.

Another carer suggested having more supported accommodation.

It’s actually quite hard to get the right support. and so, in a way, having one on one support is good. It gives you choice and control. And it’s better than nothing because there is nothing else but a really good institution that was working well. And I don’t mean institutionalizing people. I mean, having them living in supportive accommodation, ideally in a little unit of their own, where there’s 24/7 support, like the [name] foundation is trying to achieve. – P2 Carer.

Still another carer indicated that service users should not be made to feel as though their illness was their fault.

The moment you start making him feel that his problems are bad or his fault, or he’s not doing enough, I think that’s one of the biggest barriers to him in recovery and accessing services. So the ideal model … [would be] … He’s in control. He’s not made to feel bad, he’s not done something wrong, that he failed. It’s always got to be that positive, you know, like, that sandwich effect. You really need to use that. – P4 Carer.

Respondents recommended that healthcare professionals needed to respect clients and carers, and take them seriously.

I’m more just hoping the services can understand that people only contact them when they just can’t manage any more themselves, when they really are desperate to keep their loved one alive. So, when they’re contacted, they need to take in everything that person says or all the reports they have from the past and read it and act on it. Because they’ve actually got the authority that can help someone change their life. Because if I took him there with a heart attack, there was no way they’d say “He’s feeling fine”. – P6 Carer.

Still others mentioned that the mental health system and training of psychiatrists needed updating to include consumer needs.

I used to be very critical of doctors, [and] psychiatrists, but I just think the system hasn’t been supporting them either. But that also their training is outdated. It’s not just your chemistry, if someone’s been unwell for 20 years, and they haven’t got a friend, they haven’t got a job, they’ve lost their confidence, they’re still battling anxieties, and lack of involvement in life. Someone who knows their chemistry, but isn’t able to tap into your psychosocial capacity building, and isn’t able to discuss what’s really needed…. – P2 Carer1.

## 4 Discussion

This study explored the views and experiences of service users and carers on the services they used for their mental health challenges. The findings point to three key components of mental health service design. The first component relates to enabling service access. The second component relates to mental health service structures and the third to delivery of care. The findings also highlight participants views on possible causes of the shortcomings that plague mental health services.

### 4.1 Shortcomings in enabling service access

The findings from this study that relate to improving service access include expanding the type of services to include groups with unique needs. Participants described how there were no services available for transgender individuals and those, who owing to their symptoms, refused to leave their residence and remained isolated. Since the health system depends on people actively seeking help from services, this did not help those for whom, their mental illness itself prevented them from reaching out for help. Reports of home-based services have been described for older people (33) and women with schizophrenia (34). However, the challenges of initial engagement with services for mentally ill individuals who are isolated at home requires further research. The lack of carer involvement in the care of such individuals might be a missed opportunity for mental health services as they are an essential resource for mental health service provision (11).

Participants also reported that there was a lack of mental health services for families. Having both a parent and child from a family with mental health challenges appears to be a more challenging prospect for service delivery. Previous studies have reported that family members experienced a lack of confirmation and cooperation from professionals and felt powerlessness and alienated in the care being provided (35). However, some argue that confidentiality in terms of information sharing and decision-making remains contentious at a practice level (36). Younger practitioners believe that organizational factors are more significant barriers to family involvement (37). Implementation of family mental health services would require a cultural and organizational shift towards working with families and the establishment of working routines that facilitate family involvement (38). Further research into effective strategies for working with families is warranted (36).

Improving access also requires improving the type and reach of information on services available. A lack of information on community supports was another finding that respondents highlighted particularly for those who were discharged from hospital. In developed countries, the lack of information on services has not been considered a major barrier to access. However, in this case, consumers who are discharged from hospital before they felt ready were seeking specific information on where they could continue to receive high care. Whether or not this was an isolated issue was beyond the scope of this study.

### 4.2 Shortcomings in mental health service structures

Our findings that related to shortcomings in mental health service structures include fragmentation of services, and a lack of integration with other services and stakeholders. Participants reported that services were fragmented and operated in isolation. The persisting gaps and lack of integration in the system is a well-known shortcoming of mental health services (16, 39). However, this is not unique to Australia. Studies from Canada (40) and Europe (41, 42) have also highlighted the issue and challenges to integrated care such as the lack of funding, inadequate human resources, difficulties in access and the lack of availability of relevant treatments, appear to be similar in many developed countries (41). Healthcare professionals have previously cited issues such as affordability and accessibility, as well as the issue that the model of care is crisis-driven (39). It has also been previously reported that those who were not acutely ill found it almost impossible to secure inpatient care (43). Although these issues were first reported about three decades ago, little has changed on the ground (44).

The finding that GPs were not considered part of the mental health team is interesting. The need for GPs to be an integral part of the mental health team has been identified previously (45). There are several possible reasons for this. First, mental health professionals may not consider the role of the GP as a crucial element of mental health care. Second, some GPs themselves may not have an interest or the expertise in being part of the mental health care of the service user. And finally, there may not be clear guidelines on who needs to be part of the mental health care team although the mental health treatment plan [MHTP] is written by the GP. However, people talk to their GP about their mental health problems more than any other issue (46) and the majority of mental health services in Australia are delivered by GPs (47).

Participants highlighted the need for more supported accommodation as part of improving the mental health system. Supported accommodation can take three forms. They can be 24 h staffed residential care, day-staffed residential places or lower supported accommodation (48). However, there is little evidence on the cost-effectiveness of these types of residential care (48). There are nonetheless, reports of a quiet ‘reinstitutionalisation’ in some countries with an increasing number of forensic beds and beds in the private sector (49). Some suggest that the motivation for this increase in beds might be in response to Penrose’s law (50, 51) that suggests a possible inverse relationship between psychiatric bed numbers and prison population rates (51). Irrespective of the motivation behind this expansion, the need for more supported accommodation is increasingly being recognised.

Barriers to accessing the NDIS for persons with a psychosocial disability has come under increased scrutiny on account of its complex application process, difficulty in finding supports (14, 52) and reports that the Scheme sometimes made things worse rather than better for people experiencing disability associated with mental illness (53). Addressing these issues requires intervention both within individual funding schemes and the broader policy environment (54).

### 4.3 Shortcomings in the delivery of care

Our findings related to delivery of care include providing person-centred care and engaging with consumers and carers. Participants stated that services were not providing person-centred care. Gask and Coventry affirm that person-centred care involves striving to understand the person as a whole by listening to what they say, identifying important cues to the nature of their problems and responding to their needs (55). They further argue that this requires more meaningful engagement with families and communities in the delivery of health care which will in turn need a modification of the way services and organizations work (55).

Service users and carers indicated that individuals were often discharged even when they reported being too unwell to go home. Studies on early discharge have reported that professionals and service users were positive about early discharge although service users wanted peer support (56) or transitional interventions with bridging components (57) and carers preferred hospital care that perhaps indicated their need for respite (56). Economic pressures contribute to increasing pressure to discharge people from hospital early (56).

Some argue that the main obstacle to achieving person-centred care relates to concerns of healthcare professionals about risk and decision making capacity of service users (58) while others have reported that staff (mental health nurses) often feel helpless when faced with individuals presenting with severe mental illness and their interactions with them were influenced by the workplace culture, education and training as well as concern for their own safety (59).

Participants reported that they were treated poorly by services and healthcare professionals. Previous studies have indicated that service users and carers clearly expect to receive professional healthcare; they value professionalism as well as respect and compassion (60). They also expect to have positive relationships with the healthcare professionals who care for them (60). However, they often report encountering negative attitudes and discrimination from mental health staff (61, 62). Similarly, previous reports have shown that carers’ views are also rarely solicited by mental health professionals or considered during decision making (11, 63).

Participants with BPD complained that they felt humiliated when they were accused of attention seeking. Some argue that this is a misconception among mental health professionals (64). Individuals with BPD in Australia and their carers have reported that services did not meet their needs in terms of availability and cost (65). Difficulties in accessing services for BPD have been attributed to structural factors such as the uncertainty whether BPD is a legitimate mental illness, and perceptions that BPD is an untreatable condition. Individuals with BPD are therefore often denied evidence-based treatment (66).

Carers are an essential resource for mental health service provision. They are also a rich source of expertise through experience (11). When carers are not satisfied with the way they are treated by mental health services, they become reluctant to seek help from them (67). Strategies to support carer participation are urgently required if the goals of state and national mental health policy in Australia are to be realized (68). The World Psychiatric Association [WPA] has made recommendations on best practices when working with service users and family carers (69). However, improving partnerships with carers of people with mental illness will require significant leadership from mental health services to support shifts in organizational culture and practice (70). A more nuanced understanding of confidentiality is also required to overcome the barriers to involving family carers more meaningfully in care (71).

### 4.4 Causes of the shortcomings

Participants in our study were of the opinion that services are overloaded and under resourced. This is a long-standing issue. A recent study from the state of South Australia showed that there were an estimated 26,810 people living with severe mental illness per annum who required psychosocial support. Of those, 3,200 people were receiving support through the NDIS and a further 4,489 from all other state and Federally funded programs. That means there were about 19,000 persons with an unmet need for services (72). One participant opined that psychiatry training needed to be updated to include being able to identify and help address unmet needs of consumers. Opinions on the need for a review of psychiatry training have started emerging (73, 74). However, they do not necessarily focus on identifying and addressing unmet needs of consumers, which when met, lead to better overall outcomes (8).

Mental health reform is promised in policy documents. However, implementation is difficult. In Australia, funding for mental health services is shared between the federal, state and territory governments as well as individuals and private health insurers (75). However, National policies do not necessarily align between the federal and state funding structures thereby preventing their implementation (76). A 2023 report of the Royal Australian New Zealand College of Psychiatrists refers to the mental health system as being ‘poorly funded’, ‘neglected’ and ‘operating in crisis’ where individuals find it hard to access services often because they are unaffordable (77). There are ongoing calls for reform and increased investment in the Australian mental health care system (39).

The small number of service user participants (5) is perhaps a limitation of this study. However, the number of carers who could provide trustworthy information on behalf of the individuals they cared for, making up a total of 20 participants was considered adequate. Furthermore, the focus of this paper was depth rather than breadth where consumers and carers were able to describe how the shortcomings of the mental health system were barriers to recovery.

## 5 Conclusion

Participants in this study identified several shortcomings with the public mental health system in Australia for persons with severe mental health challenges. The findings point to three key components of mental health service design. The first component relates to service access that excludes groups with unique needs such as those who remained isolated at home, transgender individuals and families where more than one member has a mental illness. The second component relates to mental health service structures and includes fragmentation of services and a lack of integration with other services and stakeholders. The third component relates to the delivery of care which includes the lack of person-centred care and poor engagement with consumers and carers. Participants were of the opinion that most of the shortcomings of services were due to being overloaded and under resourced. This paper highlights some possible themes for further exploration with larger and more diverse samples.

## Data availability statement

The original contributions presented in the study are included in the article/supplementary material. Further inquiries can be directed to the corresponding author.

## Ethics statement

The studies involving humans were approved by Monash University Human Research Ethics Committee. The studies were conducted in accordance with the local legislation and institutional requirements. Written informed consent for participation in this study was provided by the participants’ legal guardians/next of kin.

## Author contributions

AI: Conceptualization, Data curation, Formal analysis, Methodology, Project administration, Writing – original draft, Writing – review & editing. CL: Data curation, Formal analysis, Writing – review & editing. SL: Data curation, Formal analysis, Writing – review & editing. AD: Writing – review & editing.
